# Geographic Regional Variation in Patterns of Racial and Ethnic Disparities in Late-Life Depression

**DOI:** 10.1093/geroni/igaa057.545

**Published:** 2020-12-16

**Authors:** Chirag Vyas, Charles Reynolds, David Mischoulon, Grace Chang, Olivia Okereke

**Affiliations:** 1 Massachusetts General Hospital, Boston, Massachusetts, United States; 2 University of Pittsburgh Medical Center, Pittsburgh, United States; 3 VA Brockton Healthcare System, Brockton, United States

## Abstract

There is evidence of racial/ethnic disparities in late-life depression (LLD) burden and treatment in the US. Geographic region may be a novel social determinant; yet, limited data exist regarding the interplay of geographic region with racial/ethnic differences in LLD severity, item-level symptom burden and treatment. We conducted a cross-sectional study among 25,503 men aged 50+ years and women aged 55+ years in VITAL-DEP (VITamin D and OmegA-3 TriaL-Depression Endpoint Prevention), an ancillary study to the VITAL trial. Racial/ethnic groups included Non-Hispanic White, Black, Hispanic, Asian, and other groups (Native American/Alaskan Native and other/multiple/unspecified-race/ethnicity). We assessed depression status using: the Patient Health Questionnaire-8 (PHQ-8); self-reported clinician/physician diagnosis of depression; medication and/or counseling treatment for depression. In the full sample, Midwest region was significantly associated with 12% lower severity of LLD, compared to Northeast region (rate ratio (RR) (95% confidence interval (CI)): 0.88 (0.83-0.93)). However, racial/ethnic differences in LLD varied by region. For example, in the Midwest, Blacks and Hispanics had significantly higher depression severity compared to non-Hispanic Whites (RR (95% CI): for Black, 1.16 (1.02-1.31); for Hispanic, 2.03 (1.38-3.00)). Furthermore, in multivariable-adjusted logistic regression models, minority vs. non-Hispanic White adults had 2- to 3-fold significantly higher odds of several item-level symptoms across all regions, especially in the Midwest and Southwest. Finally, among those endorsing PHQ-8≥10, Blacks had 60-80% significantly lower odds of depression treatment, compared to non-Hispanic Whites, in all regions. In summary, we observed significant geographic variation in patterns of racial/ethnic disparities in LLD outcomes. This requires further longitudinal investigation.

